# First endoscopic submucosal dissection of gastroesophageal junction carcinoma in a 72-year old male from Saudi Arabia

**DOI:** 10.1016/j.ijscr.2019.02.034

**Published:** 2019-03-05

**Authors:** Abdullah AlShammari, Sreyoshi Fatima Alam, Mohamed Khan, Mohammad Aburahmah

**Affiliations:** a College of Medicine, Alfaisal University, P.O. Box 50927, Riyadh, 11533, Saudi Arabia; b King Faisal Specialist Hospital and Research Center (KFSH&RC), P.O. Box 3354, Riyadh 11211, Saudi Arabia

**Keywords:** Endoscopic submucosal dissection, Gastroesophageal junction, Adenocarcinoma, Case report

## Abstract

**Introduction:**

Esophageal and gastric tumors are very lethal malignancies. Their most common histology is adenocarcinoma for more than 90% of all gastroesophageal tumors.

**Case presentation:**

Herein, we report a case of endoscopic submucosal dissection for gastroesophageal junction carcinoma, the first of its kind to be performed in the Kingdom of Saudi Arabia for a 72-year-old patient, who was found to have a gastroesophageal junction mass extending to the cardia. Complete excision was performed via endoscopic submucosal dissection. There were no complications in the postoperative period and the patient was discharged in good condition.

**Discussion:**

Over time, the management of gastroesophageal junction carcinoma has evolved along with the surgical approach. Endoscopic submucosal dissection preserves the anatomical structures and not only leads to a better quality of life, but also decreases morbidity and mortality. The procedure shows favorable results for early-stage gastroesophageal junction carcinoma as a treatment option with excellent en bloc resection rates.

**Conclusion:**

Endoscopic submucosal dissection is an acceptable management for T1 gastroesophageal junction carcinoma

## Highlights

• Esophageal and gastric adenocarcinoma account for more than 90% of all gastroesophageal tumours.
• We report a case of endoscopic submucosal dissection for gastroesophageal junction carcinoma (GEJ).
• This case is the first of its kind to be performed in the Kingdom of Saudi Arabia.
• The management of gastroesophageal junction carcinoma has evolved along with the surgical approach.
• Endoscopic submucosal dissection shows a superior treatment method as en bloc resection for early-stage GEJ carcinoma.

## 1 Introduction

Carcinomas of the upper gastrointestinal (GI) tract are highly lethal malignancies. Locally advanced unresectable and metastatic tumours are not curable. The goals of therapy include symptomatic palliation and survival prolongation, which can be most effectively achieved via systematic treatment. Carcinoma of the gastroesophageal junction (GEJ) is now classified as a separate entity with a distinct pathophysiological profile [1]. Herein, we describe a case of GEJ carcinoma extending into the cardia, it was diagnosed via endoscopic ultrasound and subsequently treated by endoscopic submucosal dissection (ESD) for the first time in Saudi Arabia. The work has been reported in line with the SCARE criteria [2].

## 2 Case presentation

A 72-year-old healthy male presented with mild, intermittent, non-radiating, epigastric pain, that was associated with nausea and vomiting. Pain was exacerbated by food intake and relieved by fasting. The rest of the history was unremarkable. Physical examination findings were unremarkable with no evidence of palpable abdominal mass.

**Fig. 1:**
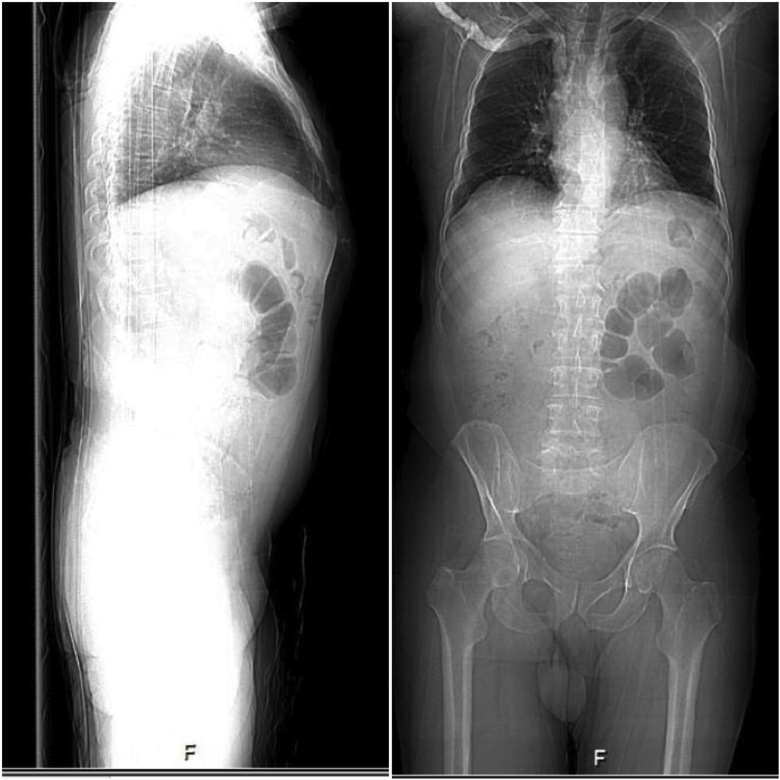
A Soft tissue heterogeneous polypoidal mass located in the GEJ, which is extending to the gastric cardia measuring 1.3 × 2.7 × 2.5 cm with no evidence of lymphadenopathy or metastasis.

**Fig. 2:**
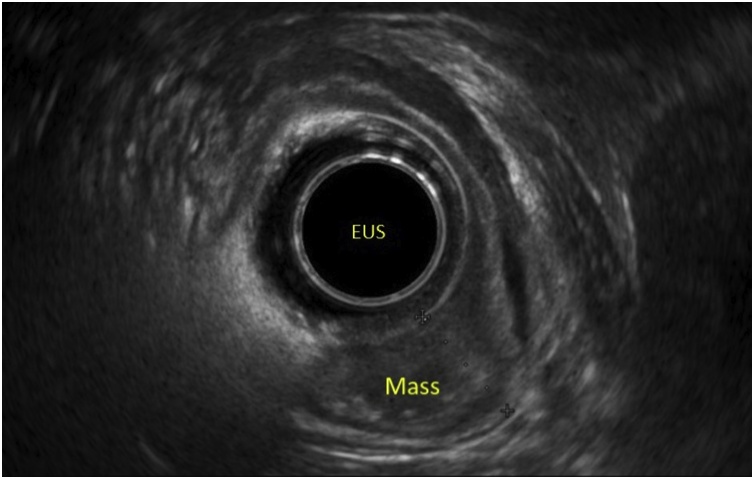
Endoscopic US showing T1 mass in the gastroesophageal junction.

PET-CT scan revealed a mass located in the GEJ extending to the cardia with heterogeneous thickening. The study also showed normal mediastinal structures with no evidence of lymphadenopathy or metastasis (Fig. 1). Endoscopic Ultrasound showed a T1 mass (Fig. 2) and the pathology report for the endoscopic biopsy indicated a low-grade intestinal type adenocarcinoma. The mass was staged as T1 GEJ Carcinoma (T1N0M0).

**Fig. 3:**
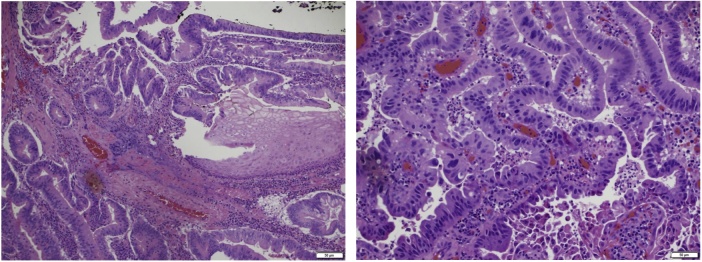
Adenocarcinoma, intestinal type, low grade.

**Fig. 4:**
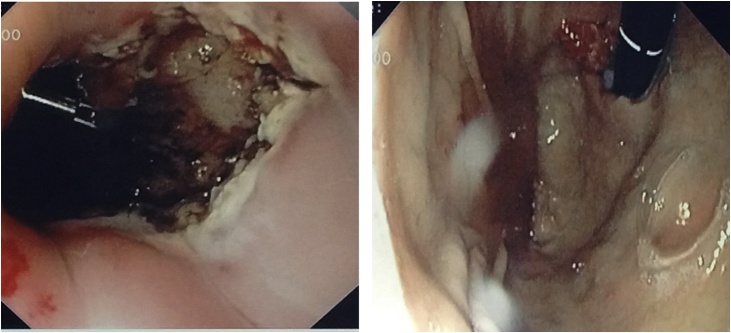
Intraoperative pictures of the mass before and after dissection.

On the 22nd of May, 2016, the patient underwent endoscopic submucosal dissection, the first of its kind to be performed in Saudi Arabia. There were no other lesions, suspicious masses or hiatal hernia. Dissection started from the proximal end and advanced deep within and below the mucosa. Then, parts of the mass were cut one by one using a circular cautery. The targeted tissues were retrieved carefully in the basket while maintaining adequate hemostasis. An intra-operative frozen section was sent to pathology that confirmed Gastric cancer intestinal type (Fig. 3) and satisfactory negative margins of each side that was taken separately. To ensure that adequate resection was achieved, and the mass was completely retrieved with negative margins, the scope was inserted multiple times and the targeted area was inspected thoroughly. As a first case, working in a retroflex position during the dissection of the distal part of the mass was our biggest challenge considering its difficult location. The procedure was carried out with minimal bleeding that was encountered and controlled. Suspicious micro-perforations were reviewed cautiously, yet, no significant adverse effects had been observed. At the 18 months follow up, no recurrence or metastasis were detected (Fig. 4).

## 3 Conclusion

In conclusion, endoscopic submucosal dissection is an acceptable management for T1 GEJ carcinoma. Further research is required for a consensus on criteria and management approach. The risks and benefits of minimally invasive approach with less morbidity and mortality versus possible long term complications have to attain an acceptable balance, and that requires a case-based review of the approach. With time, a standardization of the approach will aid physicians and surgeons to decide on the best modality and will simplify the decision-making process.

## 4 Discussion

Esophageal and gastric malignancies are a major cause of cancer-related mortality with adenocarcinoma being the most common histology (> 90%) [3]. Type II GEJ carcinomas show a predilection for males, with 4.8:1 male to female ratio. The management of esophageal cancers and gastric cancers have slight variations; hence gastroesophageal tumor management has been a point of debate [4]. In our case, the tumor was detected by ultrasound enhanced endoscopy in its early stage. This signifies the importance of endoscopic evaluation of the depth of invasion of a tumor before removal as surgery may be avoided in some cases.

The distinction between an intestinal type GEJ adenocarcinoma of esophageal and gastric origin can be made through history, principally positive for GERD, and histological findings demonstrating esophageal adenocarcinoma. Biopsy samples from the gastric antrum and corpus presented with signs of atrophy, intestinal metaplasia and body predominant gastritis, indicate a gastric origin [5]. In line with McColl, our patient showed no signs of GERD, yet his aggravating and relieving factors were in favor of a gastric origin. The tumor extended into the cardia and therefore, it required meticulous dissection and superb surgical skills.

The diagnostic workup for GEJ adenocarcinoma includes endoscopy, endoscopic ultrasound (EUS), and computed tomography (CT), with 80–90% accuracy to determine the exact tumor location and its spread to the upper mediastinal lymph nodes. For optimal nodal staging, EUS-guided FNA shows highest accuracy, yet it is not feasible in all cases due to technical difficulties. FDG-PET scans are gaining ground due to its diagnostic accuracy in detecting distant metastases [6]. Since the endoscopic dissection technique was performed for the first time in this case and an endoscopic intervention can preclude complete accessibility, we used a multimodality approach with PET-CT, EUS and EUS-guided FNA for optimal evaluation.

Over time, the management has evolved from surgical resection alone, to the addition of adjuvant and neoadjuvant therapies for locally advanced disease, since perioperative chemotherapy has shown to improve survival. Similarly, surgical options have developed ranging from transthoracic esophagectomy to total gastrectomy with extended distal esophageal resection. Comparison of the various techniques involves assessment of oncologic efficacy, perioperative outcomes, survival, and quality of life. Hence, transthoracic esophagectomy with gastric tube formation has been accepted as the surgical standard for adenocarcinoma of the distal esophagus (GEJ type I). Classically, a proximal negative margin of a minimum of 5 cm is desirable [7].

According to the US Gastric Cancer Collaborative data of 162 patients from seven USA centers (Siewert II and III cancers of the cardia or GE junction:67.6%), the proximal margin length was not associated with local recurrence or overall survival, but rather concluded that if esophagectomy is required, efforts to achieve a specific proximal margin should not be undertaken [7]. On the other hand, studies have also shown that margins depend on several factors such as histological type, the presence of Barrett’s esophagus, T stage, and lymph node metastases [8,9]. Although, it is not common to find a patient with T1 stage gastric adenocarcinoma in Saudi Arabia based on our observation, it was a good opportunity to try giving our patient the standard procedure by a trained performer under general anesthesia. Keeping in mind that converting to laparoscopic total gastrectomy and distal esophagectomy with Roux-en-Y anastomosis and D1 lymph node dissection was fully explained to the patient in case of perforation, major bleeding that can't be controlled, difficulty to complete the procedure safely or if we did not achieve negative margins.

A systematic review and meta-analysis stated that ESD has been recognized as a preferred treatment for differentiated gastric adenocarcinoma without ulceration (T1a) with a diameter of ≤2 cm [10]. Accordingly, a better quality of life for patients is achieved via ESD as a non-invasive procedure with decreased morbidity and mortality. The study showed that ESD in early stage GEJ carcinoma is a feasible treatment option with excellent en bloc resection rates. The complete resection rate of ESD in early stage GEJ carcinoma was 87.0% (95% CI 79.0–92.0%). No local or distant metastases were observed in the 269 patients who met the curative resection criteria suggesting favorable long-term results. Contrariwise, of the 90 patients who underwent noncurative resection, 3 (3.3%) had local recurrence and 2 (2.2%) had distant metastasis. The study concluded that large-scale data are necessary to form a definite conclusion, but in the meantime, curative resection criteria for gastric cancer can be used for GEJ carcinoma [10]. Given the parameters of this case, a successful endoscopic submucosal dissection was achieved which provided our patient with a better quality of life on the long run.

## Conflicts of interest

No conflicts of interest.

## Funding

No source of funding.

## Ethical approval

As the report is case report a waiver was obtained from the Office of Research Affairs at KFSH.

## Consent

Written informed consent was obtained from the patient for publication of this case report and accompanying images. A copy of the written consent is available for review by the Editor-in-Chief of this journal upon request.

## Author contribution

Abdullah AlShammari: design, writing and reviewing the study.

Fatima Alam: searching the literature and data collection.

Moahmmad Khan: writing and reviewing the article.

Mohammed AbuRahma: design and review the article.

## Registration of research studies

Not applicable.

## Guarantor

Abdullah AlShammari.

## Provenance and peer review

Not commissioned, peer reviewed.
